# Area deprivation and its association with health in a cross-sectional study: are the results biased by recent migration?

**DOI:** 10.1186/1475-9276-6-10

**Published:** 2007-09-20

**Authors:** Fredrik Niclas Piro, Øyvind Næss, Bjørgulf Claussen

**Affiliations:** 1 Institute of General Practice and Community Medicine, University of Oslo, Norway

## Abstract

**Background:**

The association between area deprivation and health has mostly been examined in cross-sectional studies or prospective studies with short follow-up. These studies have rarely taken migration into account. This is a possible source of misclassification of exposure, i.e. an unknown number of study participants are attributed an exposure of area deprivation that they may have experienced too short for it to have any influence. The aim of this article was to examine to what extent associations between area deprivation and health outcomes were biased by recent migration.

**Methods:**

Based on data from the Oslo Health Study, a cross-sectional study conducted in 2000 in Oslo, Norway, we used six health outcomes (self rated health, mental health, coronary heart disease, chronic obstructive pulmonary disease, smoking and exercise) and considered migration nine years prior to the study conduct. Migration into Oslo, between the areas of Oslo, and the changes in area deprivation during the period were taken into account. Associations were investigated by multilevel logistic regression analyses.

**Results:**

After adjustment for individual socio-demographic variables we found significant associations between area deprivation and all health outcomes. Accounting for migration into Oslo and between areas of Oslo did not change these associations much. However, the people who migrated into Oslo were younger and had lower prevalences of unfavourable health outcomes than those who were already living in Oslo. But since they were evenly distributed across the area deprivation quintiles, they had little influence on the associations between area deprivation and health. Evidence of selective migration within Oslo was weak, as both moving up and down in the deprivation hierarchy was associated with significantly worse health than not moving.

**Conclusion:**

We have documented significant associations between area deprivation and health outcomes in Oslo after adjustment for socio-demographic variables in a cross-sectional study. These associations were weakly biased by recent migration. From our results it still appears that migration prior to study conduct may be relevant to investigate even within a relatively short period of time, whereas changes in area deprivation during such a period is of limited interest.

## Background

In studies of area effects on health, misclassification of exposure due to recent migration may be a problem. Area effects on health has mostly been examined in cross-sectional studies or prospective studies with short follow-up, but migration is rarely taken into account. Studies that do not take length of residence into account may give spurious area effects on health, because individuals may be assigned an area exposure that they may have experienced too short for it to have any effect [1], and their current health may instead show the possible effects of their previous areas of residence [2], e.g. as in Blakely et al[3], who suggested that for people aged 45 years and older, income inequality up to 15 years previously may be more strongly associated with self rated health than income inequality measured contemporaneously. A cross-sectional design appears inappropriate when thinking about socially and biologically plausible causal pathways by which areas might influence health [4].

In a cross-sectional study of a city population several types of migration may bias the results. Firstly, the aspect of selective migration, i.e. people in poor (or good) health moving to areas of poor (or good) status. Secondly, the aspect of health-promoting or health-damaging effects of the act of moving itself. For example, Kahlmeier et al[5] found an increased wellbeing of movers who had moved into areas with improved environmental housing quality. On the other hand, some people may have difficulties in adapting to a new area, e.g. in creating connections with neighbours, which may be detrimental to health. The common denominator in dealing with these two types of migration is the requirement for longitudinal health data. Therefore, these issues will remain potential sources of bias in cross-sectional studies.

Still, some aspects of migration may be dealt with even in cross-sectional studies, in which we can imagine two types of migration prior to the study conduct: migration into the city and within the city. Both need to be considered. This can be done by using retrospective linkage of residence data. But because some people tend to have rather frequent changes in residence, we believe it is important to have retrospective annual data of residence in order to capture the true exposure for the study participants. Furthermore, areas themselves may change over time, implying that people may experience different levels of area deprivation without moving at all. Boyle et al[6] addressed these problems using a selection of people from the ONS Longitudinal Study for England and Wales that had not moved between 1971 and 1991, and who were living in non-deprived households throughout the period. The authors found that changes in deprivation in the residential area had a demonstrable effect on morbidity. The study also showed a clear gradient for morbidity which demonstrated that people living in areas which remained most deprived throughout the period had the most morbidity, and people living in areas which remained least deprived had the lowest morbidity. This is in accordance with a number of studies measuring area effects cross-sectionally with a variety of different measures; self rated health [7-12], mental health [13,14], coronary heart disease [15], respiratory disease [8,16,17], daily smoking [8,17-22] and exercise [18,23,24], after adjusting for individual characteristics. Significant associations between areas and health have been widely documented [25], but potential bias from migration is rarely investigated. Some studies with large study populations have more or less eliminated migration bias by restricting their analyses to individuals who have resided in a given area over a relatively long period [26], whereas other studies with smaller populations have adjusted for number of years in current area of living [27]. Most studies neglect the aspect of migration, presumably in lack of data.

The aim of this study was to investigate to what extent cross-sectional analyses of the associations between area deprivation and health (self rated health, mental health, coronary heart disease, chronic obstructive pulmonary disease, smoking and exercise) are biased by recent migration. We specifically tested whether: 1) the associations between area deprivation and health were different if we removed those participants that had recently migrated into Oslo (i.e. testing bias from migration *into *Oslo), and 2) whether the associations between area deprivation and health were different if those who had lived in Oslo in the period 1992–2000 were assigned a deprivation measure taking all years of residence into account, instead of only year 2000 (i.e. bias from migration *within *Oslo).

### Area deprivation

Area deprivation is a frequently used concept but has no singular definition [25]. Anderson et al[28] claim that area deprivation "may summarise an area's potential for health risk from ecological concentration of poverty, unemployment, economic disinvestment, and social disorganisation". Our definition of the concept is much in line with this. We define area deprivation as "the clustering of people with limited possibilities for choosing destination of residence". Areas with a high level of socioeconomic disadvantage may also be disadvantaged with respect to other area characteristics in ways that influence health independently of the socioeconomic characteristics of the people living in such areas [17]. Massey [29] claims that to the extent disadvantaged individuals are concentrated in geographically defined areas, disadvantage becomes a characteristic of the areas too. Such disadvantage may take two forms [10]; physical disorder (such as abandoned buildings, noise, graffiti, vandalism, filth and disrepair) and social disorder (such as crime, loitering, public drinking or drug use, conflicts and indifference). Both types of disorder lead to unattractiveness in the housing market. Structural factors through the employment and housing market offer limited possibilities for withdrawal from the area for some people [8], who will have few options to escape from unfavourable conditions that have been found to be associated with health, e.g. poor housing quality [5,30-32] and poor physical quality of the residential environment [5,11,31,33,34].

A Norwegian study from 1994 demonstrated how certain economically disadvantaged inner-city areas of Oslo were characterised by three specific groups [35]. Firstly, a large clustering of people with several lifestyle disadvantages (e.g. unemployment, poor health). Secondly, a large group of people who had recently moved in, but who did not regard their destination of arrival as a desired area to move to. Furthermore, within these areas the largest percentage of people in Oslo who wanted to move to another area was found. And thirdly, a large group of young people, who would leave the area after a few years, regarding the area as an interim destination. In reference to the housing market; because the value of each home depends on the quality of the neighbourhood, owners have a substantial personal interest in preserving and improving it, compared to short-time renters [10]. Therefore, the extent to which young people exploit cheap tenure possibilities in low price areas, leading to high area turnover, may decrease social cohesion and induce more social disorganisation [7,17]. Overall, we argue that individual socioeconomic disadvantage restricts freedom of choosing where to live, and that those possibilities that do exist are characterized by physical and social disorder. Some support of this view was found by Stafford and Marmot [30] who demonstrated that perceived neighbourhood problems (e.g. lack of facilities, noise) and perceived housing problems mediated the association between area deprivation and poor self rated health.

## Methods

### Study population and participants

Data were obtained from the Oslo Health Study (HUBRO), a joint collaboration between the Oslo City Council, the University of Oslo, and the Norwegian Institute of Public Health in 2000–2001. A total of 40 888 persons in five age cohorts were invited to participate. Participation rate was 46% [36]. We included those aged 30 (participation rate 36.1%), 40 (participation rate 43.7%), 45 (participation rate 46.5%) and 60 (participation rate 55.4%). This left us with a total population of 14 608 persons. These were divided into three groups: Stayers (those who lived in the same area in Oslo in all years, n = 6 072), migrants (those who lived in Oslo in all years, but moved between areas, n = 4 995) and in-migrants (those who moved into Oslo during 1992–2000, n = 3 541).

### Independent individual variables

*Age*, *sex*, *marital status *(married/registered partner; unmarried; and divorced, separated, widow/widower and other marital forms), *education *(academic college or university education, and lower educational forms), and *employment status *(full-time employed, part-time employed, and not working) were self-reported. *Income *was total taxable income of 1999 (linked from Statistics Norway), quintilised with the first quintile representing the highest incomes. We included this many measures of individual socioeconomic position in order to reduce the likelihood that the associations between area deprivation and health were attributable to unmeasured or poorly measured confounding by individual factors [23,25,37].

### Independent variable: Area deprivation

Oslo is the capital city of Norway and the country's largest city. At the time of the study, the city was divided into 25 administrative areas with a total population of 503 720. Area deprivation was a composite index of five items: 1) percentage of population affected by social security benefits (i.e. both direct receivers and any family members), 2) percentage of unemployed (age 16–66), 3) percentage receiving disability pension (age 16–66), 4) percentage with no academic college or university education (age 16–66), and 5) average taxable income in the area. The items were chosen in order to reflect the clustering of people within an area with anticipated limited possibilities of choosing destination of residence, particularly those with limited exit options from current area of living. Across all five indicators, the areas were quintilised. A composite rank score from 5 (least deprived) to 25 (most deprived) was calculated and quintilised for the analyses. This was repeated for all years back to 1992, i.e. we had annual area deprivation indexes throughout the period 1992–2000. Data were provided by the Oslo City Council.

### Dependent variables: Health outcomes

*Self rated health (SRH) *was derived from the question: "How would you describe your present state of health (poor, not very good, good, very good)", dichotomised into poor/not very good and good/very good. *Mental health (HSCL) *was measured by The HSCL-10 consisting of 10 items on a 4-point scale [38]. Cut-off was set at 1.85 (< 1.85 indicating good mental health, and > 1.85 indicating poor mental health), which has been found equivalent to the conventional cut-off point at 1.75 for HSCL-25, and is a validated measure of mental health [39]. *Coronary heart disease (CHD) *was measured by The Rose Questionnaire of angina pectoris [40]. *Chronic obstructive pulmonary disease (COPD) *was assessed by a modified version of the Medical Research Council's questionnaire with three items [41]. *Smoking *was assessed by the question "Have you smoked or do you smoke?" which has been shown to produce relatively reliable information about smoking habits [42]. Former and never smokers were collapsed into one group, contrasted to current smokers. *Exercise *was measured by the question "What kind of physical activity have you undertaken in the course of the past year? Estimate a weekly average for the year (hard physical activity, you sweat or feel out of breath)". The variable was dichotomised into no exercise at all and have exercised. Measuring exercise by self-reported data on number of occasions or time spent on such activities is commonly used [18,24,43,44]. Missing values on the health outcomes were 132 for SRH, 579 for HSCL, 111 for CHD, 1 940 for COPD, 107 for current smoking and 924 for no exercise. Data on COPD was obtained from an additional questionnaire that had a much lower response rate than the main questionnaire in the Oslo Health Study. Hence the large number of missing values for COPD.

### Statistics

We specified three binomial multilevel logistic regression models, estimating the parameters by the penalised quasilikelihood method. Analyses were conducted for all health outcomes, in which we measured the association between area deprivation and health after adjusting for individual variables. Model 1 included all HUBRO-participants, i.e. a traditional cross-sectional analysis. In model 2 we removed the in-migrants, and in model 3 area deprivation was measured as an average of all areas of residence during the period 1992–2000 for those who had lived in Oslo in all years, i.e. a so-called multiple membership design [45]. Between-area variance was calculated, indicating the unexplained variance in the outcome of interest in the 25 areas. The results of the regression analyses are presented as odds ratios (95% confidence intervals) with test for trend estimates for area deprivation, which represent the odds ratios when the are deprivation quintiles are analysed as a continuous variable. Analyses were carried out using the software MLwiN (version 1.10.007) [46]. SPSS for Windows (version 11.0) was used to perform χ^2 ^tests. SAS (version 9.1) was used to perform Cochran-Armitage trend tests.

### Missing respondents

The Oslo Health Study had a large proportion of missing respondents (54%). Compared to the invited population, the following subgroups were under-represented [36]: males, the youngest age-group, unmarried and separated/divorced, those not born in Norway, inner city dwellers, those with unknown or lower secondary education, low income groups, and receivers of disability benefits. But self-selection by socio-demographic background did not influence prevalence estimates in SRH, HSCL and smoking to any degree [36]. To what extent CHD, COPD and exercise were influenced, we cannot tell. Furthermore, 12.3% in our sample had one or more missing values on the independent variables (Table 1). Those with one or more missing values were worse off in terms of both socio-demographic variables and health outcomes than those with no missing values (figures not shown). In order to avoid bias from exclusion of participants with missing values, we included them as separate missing categories in the analyses.

**Table 1:** Study population across stayers/migrants (S/M)^1 ^and in-migrants (I-M)^2^. Percent distribution, and prevalences.

	**Percent**		**SRH**		**HSCL**		**CHD**		**COPD**		**Smoking**		**No exercise**	
	**S/M**	I-M	**S/M**	I-M	**S/M**	I-M	**S/M**	I-M	**S/M**	I-M	**S/M**	I-M	**S/M**	I-M
Age*														
30	**17.4**	57.3	**13.8**	10.3	**9.6**	7.4	**4.1**	2.4	**2.5**	2.1	**25.7**	19.6	**48.0**	46.2
40	**24.1**	18.1	**19.6**	23.0	**12.1**	14.4	**5.1**	6.9	**3.7**	3.3	**31.0**	31.5	**55.2**	59.0
45	**23.5**	12.0	**21.3**	25.6	**12.0**	16.4	**5.0**	9.6	**5.1**	7.3	**34.1**	32.9	**53.6**	55.8
60	**35.0**	12.6	**32.1**	28.8	**12.5**	11.3	**10.0**	12.6	**5.7**	8.0	**25.8**	23.3	**63.9**	65.2
Sex*														
Women	**55.0**	53.3	**25.4**	17.9	**14.2**	11.8	**7.2**	5.4	**4.0**	3.1	**29.4**	22.5	**58.5**	53.9
Men	**45.0**	46.7	**20.8**	15.2	**8.8**	8.4	**6.0**	5.2	**5.3**	4.0	**28.4**	25.3	**53.9**	49.3
**Marital status**														
Married	**52.8**	38.4	**23.7**	21.9	**9.3**	10.5	**6.8**	7.6	**4.2**	3.0	**23.5**	18.1	**58.5**	58.6
Unmarried	**29.2**	48.9	**16.8**	9.4	**11.6**	7.8	**3.9**	2.0	**3.7**	2.6	**32.1**	25.4	**51.1**	44.8
Divorced	**17.9**	12.4	**33.1**	29.2	**19.4**	19.1	**10.5**	11.4	**7.1**	8.9	**39.9**	34.7	**59.7**	60.2
Missing	**0.1**	0.3												
**Education**														
Higher	**41.3**	43.2	**13.9**	8.2	**7.7**	5.9	**3.3**	1.7	**3.1**	2.4	**18.3**	17.4	**48.5**	41.9
Lower	**54.1**	24.8	**28.4**	20.4	**14.2**	12.9	**8.4**	7.9	**5.7**	5.8	**37.5**	36.7	**61.5**	61.4
Missing	**4.6**	32.0												
**Employment**														
Full-time	**69.0**	72.2	**15.0**	10.9	**7.4**	6.7	**4.3**	3.3	**3.7**	2.8	**28.1**	22.5	**53.2**	47.5
Part-time	**13.5**	10.3	**26.6**	21.3	**12.3**	13.9	**6.5**	5.9	**4.1**	4.8	**26.1**	25.0	**59.9**	60.2
Unemployed	**15.9**	14.8	**54.7**	39.3	**30.1**	25.0	**15.8**	13.6	**9.0**	6.9	**35.4**	30.5	**67.6**	68.1
Missing	**1.6**	2.7												
**Income**														
1st quintile	**20.9**	14.8	**11.8**	8.7	**5.2**	5.1	**3.3**	2.3	**3.1**	2.4	**21.1**	16.3	**50.2**	46.1
2nd quintile	**20.0**	17.8	**18.2**	10.1	**9.3**	8.4	**5.2**	3.5	**4.4**	2.8	**28.0**	22.6	**52.3**	40.8
3rd quintile	**19.9**	18.1	**22.2**	13.5	**10.5**	7.8	**5.3**	3.6	**4.3**	4.6	**31.5**	24.5	**56.0**	49.4
4th quintile	**20.2**	17.2	**29.7**	19.6	**14.3**	11.7	**8.8**	7.2	**4.7**	3.4	**33.3**	28.1	**61.5**	56.8
5th quintile	**19.0**	20.8	**36.2**	28.4	**21.1**	18.3	**11.0**	9.2	**6.8**	4.3	**31.5**	27.8	**63.5**	64.4
Missing	**0.0**	11.3												
Area deprivation*														
1st quintile	**22.5**	21.1	**15.1**	12.9	**7.8**	7.8	**4.0**	4.1	**3.1**	2.4	**21.1**	19.5	**50.2**	48.1
2nd quintile	**16.4**	19.2	**17.0**	10.3	**9.9**	7.0	**4.7**	2.3	**3.9**	3.4	**25.2**	21.9	**51.4**	45.3
3rd quintile	**20.4**	17.6	**25.6**	18.7	**12.1**	10.2	**7.0**	4.6	**4.7**	2.1	**32.0**	23.8	**57.9**	51.1
4th quintile	**22.0**	19.8	**27.6**	20.2	**13.8**	10.5	**7.4**	7.7	**4.9**	4.3	**32.5**	21.9	**60.9**	55.6
5th quintile	**18.7**	22.3	**31.4**	20.9	**15.7**	15.2	**10.2**	7.7	**6.6**	5.2	**34.5**	31.2	**62.1**	58.2

^1 ^Participants who resided in Oslo during 1992–2000, n = 11067.

^2 ^Participants who moved into Oslo during 1992–2000, n = 3541.

* No missing.

### Ethics and approvals

All participants of the Oslo Health Study gave their written consent. The Norwegian Data Inspectorate approved the study, the Regional Committee for Medical Research Ethics evaluated it, and it was conducted in full accordance with the World Medical Association Declaration of Helsinki.

## Results

The 25 administrative areas of Oslo were ranked according to the area deprivation index in 1992 and 2000 (Table 2), and the rankings were almost completely stable. There was a clear relationship between area deprivation and unfavourable health outcomes, indicating strong differences in health between the areas of Oslo. For example, the prevalence of poor SRH varied between 12.6% (Ullern, deprivation category 1) and 32.9% (Romsaas, deprivation category 5), indicating almost 2.4 times as high prevalence in the area with the highest compared to the lowest prevalence. This proportion was 2.6 in HSCL, 5.3 in CHD, 4.9 in COPD, 2.4 in current smoking and 1.7 in no exercise.

**Table 2:** Areas of Oslo (population, area deprivation index 1992 and 2000, and prevalences) (n = 14608).

	**Population 2000^a^**	**Index 1992**	**Index 2000**	**SRH**	**HSCL**	**CHD**	**COPD**	**Smoking**	**No exercise**
**Area**									
Vinderen	19 612	1	1	13.8	7.5	2.3	2.3	15.8	42.7
Ullern	26 607	1	1	12.6	7.4	3.9	3.6	21.4	49.8
Roea	21 310	1	1	15.6	9.5	4.3	4.0	21.2	51.6
Nordstrand	17 349	1	1	15.3	7.3	5.3	1.6	20.7	54.3
Sogn	15 823	1	2	16.5	7.6	2.9	3.3	19.8	46.4
Grefsen-Kjels.	17 765	2	1	17.9	8.8	4.7	4.7	20.7	47.7
Bygdoey-Frogn.	20 326	2	2	17.9	7.4	5.4	3.1	27.9	52.3
Ekeberg-Bek.	16 587	2	2	17.6	9.6	4.3	1.5	26.5	56.2
St.Hansh-Ullev.	28 259	2	2	15.0	10.0	4.1	5.2	26.8	47.7
Uranienb-Major.	23 809	2	2	15.4	10.4	5.0	4.1	26.2	49.8
Bjerke	22 821	3	3	24.2	10.7	6.1	3.2	29.9	56.0
Oestensjoe	15 433	3	3	24.2	10.0	7.4	5.8	29.9	57.3
Manglerud	12 309	3	3	23.3	11.8	7.5	3.6	34.3	58.1
Boeler	13 170	3	3	27.1	13.5	7.9	5.8	28.7	56.6
Hellerud	15 691	3	3	26.1	11.8	7.6	4.2	31.9	60.2
Soendre Nords.	31 380	4	4	27.9	14.2	7.7	4.4	30.4	60.4
Helsfyr-Sinsen	21 100	4	4	25.2	11.3	6.9	5.1	31.2	55.6
Lambertseter	10 230	4	4	22.0	14.4	5.2	3.6	34.8	53.1
Stovner	20 968	4	4	32.4	15.5	12.2	6.2	32.6	63.5
Furuset	29 290	4	4	28.8	12.8	8.4	4.3	31.6	63.7
Grünerl-Sofien.	26 663	5	5	28.8	19.2	7.9	7.4	37.8	57.0
Gamle Oslo	25 433	5	5	30.6	16.6	11.1	6.0	38.6	61.6
Grorud	17 075	5	5	30.2	12.9	9.2	6.7	29.5	61.0
Sagene-Torsh.	27 888	5	5	23.9	15.9	7.1	7.5	32.9	54.2
Romsaas	6 822	5	5	32.9	13.2	9.0	5.6	35.8	71.1
									
Total	503 720			22.7	11.7	6.7	4.5	28.7	55.3
Std. deviation				6.3	3.2	2.4	1.6	5.9	6.3

^a ^Mean: 20148, median: 20326, minimum: 6822, maximum: 31380, the interquartile range (IQR) of inhabitants: 9610. IQR was calculated as the distance between the 75^th ^percentile and the 25^th ^percentile.

Table 3 describes the characteristics of the five items forming the area deprivation index. Average taxable income numbers are somewhat biased because of changes in the tax legislation during the 1990s when legal options for reducing tax liability were reduced. Levels of people affected by social security benefits were slightly reduced between 1992 and 2000, while the percentages of people with higher education increased. Distribution of the items across areas was rather stable (as seen by standard deviation, range and interquartile range). Exceptions were income (which must be treated with much caution) and percentage of people affected by social security benefits for which the standard deviation and range decreased.

**Table 3:** Descriptive data of area deprivation items in 1992 and 2000.

	**Year**	**Mean**	**Median**	**Std. dev.**	**Range**	**Min.**	**Max**	**IQR**
								
**Percentage of people affected by social security benefits**	1992	9.5	7.8	5.9	23.3	1.3	24.6	7.8
	2000	6.4	5.7	3.9	16.6	1.8	18.4	5.0
								
**Percentage of unemployed**	1992	2.9	2.8	1.0	4.4	1.5	5.9	0.8
	2000	2.4	2.4	1.0	4.8	0.4	5.2	1.3
								
**Percentage receiving disability pension**	1992	7.4	8.0	2.7	9.6	2.9	12.5	4.6
	2000	7.5	7.6	2.7	9.0	3.2	12.2	4.8
								
**Percentage with no academic college/university education**	1992	26.8	23.0	12.2	41.8	11.7	53.5	20.1
	2000	35.1	32.2	11.9	40.7	17.6	58.3	20.6
								
**Average taxable income^a ^(€)**	1992	19690	18125	4327	18253	14604	32858	4820
	2000	35811	29085	14408	63944	23762	87706	11734

^a ^Exchange rate set at 1 € = 8.3 NOK. This rate was used for both 1992 and 2000.

In our sample, 75.8% had lived in Oslo in the period 1992–2000 and 24.2% were in-migrants, i.e. moving into Oslo during the same period (Table 1). χ^2 ^tests showed that the in-migrants were significantly younger, higher educated, more often unmarried and had lower income than those living in Oslo (p < .0001, figures not shown). In-migrants were significantly better off in all health outcomes, although differences in prevalences were small (p < .0001 for SRH, smoking and no exercise, p < .01 for CHD, p < .05 for HSCL and COPD). The groups differed somewhat by area deprivation (p < .0001), but none of the groups could in general be claimed to live in more deprived areas than the other, i.e. no significant linear association was found. Cochran-Armitage trend tests revealed that the social gradients in health according to area deprivation was significant for both groups (p < .001), except COPD for in-migrants (p > .05) (figures not shown). The prevalences were about twice as high in the most deprived compared to the least deprived areas for both groups, except for smoking and no exercise where differences were somewhat smaller.

Table 4 shows a matrix of movings within deprivation categories for stayers and migrants, i.e. those who had lived in Oslo since 1992. 54.8% resided in the same deprivation category in both 1992 and 2000, 22% had moved down in the deprivation hierarchy, while 23% had moved up. Thus 45.2% did not live in an area in the same deprivation category in 2000 as they did nine years before, and in addition, 24.2% of all study participants had migrated into Oslo. This implies that 58.4% of the HUBRO-participants are potentially being attributed an area deprivation category that they have only recently been exposed to if migration is not accounted for.

**Table 4:** Matrix of movings between areas within Oslo (n = 11067) between 1992 and 2000. Numbers^1^.

**Deprivation category 1992**	**Deprivation category 2000**					
	**1**	**2**	**3**	**4**	**5**	**Total**
**1 **(least deprived)	**1671**	550	49	142	81	2493
**2**	100	**1386**	49	177	105	1817
**3**	68	224	**961**	866	142	2261
**4**	74	230	428	**1310**	390	2432
**5 **(most deprived)	48	270	57	945	**744**	2064
						
**Total**	1961	2660	1544	3440	1462	11067

^1 ^Stayers in bold.

Table 5 shows differences between stayers and migrants (i.e. moved up or down in the deprivation hierarchy in the period 1992–2000). For all variables (and within all categories) there is roughly a '25-50-25' distribution, i.e. 25% moving down, 50% being stable and 25% moving up. By restricting the figures to those who had moved within Oslo, we found that those who moved up were significantly older, had higher education and income, and better self rated health (but did not differ in any other health outcomes).

**Table 5:** Predictors for moving downwards or upwards in the area deprivation ranking 1992–2000.

	**Stayers and migrants (n = 11067)**				**Migrants (n = 4995) – stayers excluded**			
	**Moving down**	**Stayers**	**Moving up**	**N**	**Moving down**	**Moving up**	**N**	**χ^2^**
**Age**								
30	37.9%	33.7%	28.4%	1931	57.1%	42.9%	1281	
40	20.9%	51.0%	28.1%	2666	42.6%	57.4%	1307	
45	17.7%	62.6%	19.8%	2601	47.1%	52.9%	976	
60	17.9%	63.0%	19.0%	3869	48.5%	51.5%	1431	.000
**Sex**								
Women	21.0%	56.6%	22.5%	6082	48.3%	51.7%	2642	
Men	23.4%	52.8%	23.8%	4985	49.6%	50.4%	2353	.359
**Marital status**								
Married	18.1%	62.3%	19.6%	5846	48.0%	52.0%	2203	
Unmarried	28.5%	42.5%	29.0%	3236	49.6%	50.4%	1861	
Divorced	23.3%	53.1%	23.6%	1983	49.8%	50.2%	930	.497
**Education**								
Higher	19.8%	56.3%	23.9%	4572	45.3%	54.7%	1999	
Lower	23.2%	54.4%	22.4%	5987	50.9%	49.1%	2729	.000
**Employment**								
Full-time	22.3%	54.4%	23.8%	7631	48.9%	51.1%	3478	
Part-time	17.5%	61.2%	21.3%	1495	45.0%	55.0%	580	
Unemployed	24.4%	52.4%	23.2%	408	51.3%	48.7%	838	.065
**Income**								
1st quintile	15.6%	60.8%	23.6%	2213	39.9%	60.1%	868	
2nd quintile	20.7%	55.8%	23.5%	2214	46.8%	53.2%	979	
3rd quintile	24.6%	53.2%	22.2%	2213	52.6%	47.4%	1035	
4th quintile	24.9%	52.3%	22.8%	2214	52.3%	47.7%	1056	
5th quintile	24.5%	52.2%	23.2%	2213	51.4%	48.6%	1057	.000
**Self rated health**								
Good	21.2%	55.8%	23.1%	8366	47.9%	52.1%	3700	
Poor	24.9%	52.1%	23.0%	2547	51.9%	48.1%	1220	.015
**HSCL**								
No	21.3%	55.7%	22.9%	9184	48.2%	51.9%	4064	
Yes	26.2%	49.2%	24.6%	1225	51.6%	48.4%	622	.114
**CHD**								
No	21.8%	55.1%	23.1%	10196	48.5%	51.5%	4577	
Yes	25.8%	51.9%	22.2%	724	53.7%	46.3%	348	.059
**COPD**								
No	21.5%	55.8%	22.7%	8739	48.7%	51.3%	3859	
Yes	25.2%	49.6%	25.2%	417	50.0%	50.0%	210	.706
**Smoking**								
No	20.9%	56.5%	22.6%	7767	48.0%	52.0%	3376	
Yes	24.7%	51.1%	24.2%	3167	50.5%	49.5%	1549	.103
**No Exercise**								
No (active)	21.0%	55.7%	23.3%	4401	47.3%	52.7%	1950	
Yes (inactive)	22.4%	54.9%	22.8%	5702	49.5%	50.5%	2574	.143

In a multivariate logistic regression analysis, after adjustment for age, sex, marital status, education, employment status and income, we found that for all health outcomes, those who had moved downwards had significantly worse health compared to the stayers (Table 6). But this was also the case for those who had moved upwards, although the associations were weaker. When the stayers were excluded, there were no significant differences in health between those who moved up and those who moved down. Thus, moving, regardless of direction, was associated with worse health compared to not moving.

**Table 6:** Odds ratios (95% CI) for moving up or down between 1992 and 2000^a^.

	**SRH**	**HSCL**	**CHD**	**COPD**	**Smoking**	**No exercise**
**Moving up**	1.15	1.07	1.16	1.12	1.06	1.11
	(1.08 – 1.22)	(0.99 – 1.16)	(1.06 – 1.27)	(0.99 – 1.26)	(1.01 – 1.12)	(1.05 – 1.17)
**Stayers**	1.00	1.00	1.00	1.00	1.00	1.00
**Moving down**	1.32	1.26	1.22	1.33	1.19	1.14
	(1.17 – 1.50)	(1.08 – 1.48)	(1.00 – 1.48)	(1.03 – 1.71)	(1.06 – 1.33)	(1.02 – 1.27)
						
**Model without stables**						
**Moving up**	1.00	1.00	1.00	1.00	1.00	1.00
**Moving down**	1.12	1.11	1.15	1.03	1.06	1.08
	(0.97 – 1.30)	(0.92 – 1.32)	(0.92 – 1.44)	(0.77 – 1.37)	(0.94 – 1.21)	(0.96 – 1.23)

^a ^Adjusted for age, sex, marital status, education, employment status and income.

After adjustment for age and sex, there were significant area variances in health outcomes (Table 7), both when examining all HUBRO-participants in 2000 (model 1) and when excluding in-migrants in 2000 (model 2). Area level variances did not vary much between the two models. After adjustment for socio-demographic variables, area variance changed to non-significant for HSCL, CHD and COPD, but significant area variance remained for SRH, smoking and no exercise, meaning that differences between areas in these outcomes were not fully explained by the composition of the people residing there.

**Table 7:** Neighbourhood level variance (standard error) according to six health outcomes.

	**SRH**	**HSCL**	**CHD**	**COPD**	**Smoking**	**No exercise**
						
**Model 1: All participants (2000)**						
**Age + sex**	0.165 (0.050)	0.099 (0.034)	0.165 (0.057)	0.120 (0.050)	0.082 (0.026)	0.060 (0.019)
**All variables^1^**	0.066 (0.023)	0.018 (0.011)	0.044 (0.022)	0.057 (0.032)	0.019 (0.008)	0.030 (0.011)
						
**Model 2: In-migrants excluded (2000)**						
**Age + sex**	0.167 (0.052)	0.105 (0.037)	0.159 (0.057)	0.115 (0.052)	0.086 (0.028)	0.056 (0.019)
**All variables^1^**	0.062 (0.022)	0.016 (0.012)	0.040 (0.023)	0.053 (0.034)	0.018 (0.009)	0.025 (0.010)

^1 ^Age, sex, marital status, education, employment status and income.

In multilevel logistic regression analyses investigating the associations between area deprivation and health after adjustment for socio-demographic variables, we first included all participants, i.e. a traditional cross-sectional study (Table 8). The respondents with missing values on any independent variables were included as separate missing categories. Differences in the distributions of independent variables (including health outcomes) by missing/no missing were tested with χ^2 ^tests (figures not shown), and showed that those with missing values differed on all variables (p < .0001) except sex and COPD (p > .05). The missing group had lower educational attainment, was more often unmarried, more often unemployed, and had lower income. 43.9% in the missing group, compared to 17.4% of those with no missing, fell in the lowest income category, and 8.8% and 21.2% respectively were in the highest income category. 25.2% of the missing group was in the most deprived category compared to 18.8% of the included, and 19.3% and 22.6% respectively were in the least deprived category. 33.8% in the missing group reported poor SRH compared to 20.1% in the included group, 17.6% reported poor HSCL (compared to 10.6%), 11.2% reported CHD (compared to 5.7%) and 64.4% reported that they performed no exercise (compared to 54.2%). The only outcome in which the missing group reported lower prevalence was in smoking where 23.3% of the missing group reported daily smoking compared to 28.3% among the included. In the multilevel regression analyses, missing categories were mainly associated with health outcomes (Table 8).

**Table 8:** Multilevel logistic regression analyses (odds ratios and 95% CI). All participants year 2000°.

	**SRH**	**HSCL**	**CHD**	**COPD**	**Smoking**	**No exercise**
						
**Age**						
30	1.00	1.00	1.00	1.00	1.00	1.00
40	1.75	1.60	1.45	1.56	1.67	1.34
	(1.51 – 2.03)	(1.35 – 1.91)	(1.13 – 1.86)	(1.12 – 2.16)	(1.48 – 1.88)	(1.20 – 1.49)
45	2.04	1.69	1.50	2.31	1.96	1.26
	(1.76 – 2.37)	(1.40 – 2.01)	(1.15 – 1.94)	(1.68 – 3.18)	(1.73 – 2.21)	(1.13 – 1.41)
60	2.65	1.29	2.22	2.02	1.06	1.79
	(2.29 – 3.06)	(1.07 – 1.56)	(1.76 – 2.86)	(1.46 – 2.80)	(0.94 – 1.28)	(1.60 – 2.01)
						
**Sex**						
Women	1.00	1.00	1.00	1.00	1.00	1.00
Men	0.98	0.75	1.02	1.55	1.08	0.86
	(0.89 – 1.07)	(0.66 – 0.85)	(0.87 – 1.18)	(1.28 – 1.88)	(1.00 – 1.18)	(0.79 – 0.93)
						
**Marital status^1^**						
Married	1.00	1.00	1.00	1.00	1.00	1.00
Unmarried	0.84	1.46	0.66	1.09	1.69	0.87
	(0.74 – 0.94)	(1.26 – 1.68)	(0.53 – 0.81)	(0.85 – 1.40)	(1.54 – 1.87)	(0.79 – 0.95)
Divorced	1.32	2.14	1.32	1.61	2.06	0.91
	(1.17 – 1.48)	(1.85 – 2.47)	(1.12 – 1.57)	(1.28 – 2.02)	(1.86 – 2.29)	(0.82 – 1.02)
						
**Education**						
Higher	1.00	1.00	1.00	1.00	1.00	1.00
Lower	1.48	1.38	1.68	1.39	2.42	1.43
	(1.33 – 1.64)	(1.21 – 1.58)	(1.39 – 2.04)	(1.11 – 1.72)	(2.22 – 2.67)	(1.32 – 1.55)
Missing	2.45	1.89	2.55	1.24	1.33	1.92
	(2.08 – 2.88)	(1.54 – 2.32)	(1.98 – 3.29)	(0.84 – 1.84)	(1.13 – 1.56)	(1.65 – 2.23)
						
**Employment**						
Full-time	1.00	1.00	1.00	1.00	1.00	1.00
Part-time	1.88	1.60	1.32	1.22	0.94	1.16
	(1.65 – 2.15)	(1.34 – 1.90)	(1.05 – 1.66)	(0.90 – 1.66)	(0.83 – 1.07)	(1.04 – 1.31)
Unemployed	4.85	3.93	2.70	2.05	1.20	1.37
	(4.29 – 5.47)	(3.38 – 4.56)	(2.24 – 3.24)	(1.59 – 2.64)	(1.06 – 1.35)	(1.22 – 1.55)
Missing	3.60	5.24	3.14	1.60	0.66	1.72
	(2.70 – 4.81)	(3.50 – 7.85)	(2.15 – 4.60)	(0.72 – 3.54)	(0.46 – 0.92)	(1.14 – 2.61)
						
**Income**						
1st quintile	1.00	1.00	1.00	1.00	1.00	1.00
2nd quintile	1.42	1.57	1.43	1.30	1.25	0.90
	(1.20 – 1.67)	(1.25 – 1.97)	(1.07 – 1.90)	(0.93 – 1.81)	(1.09 – 1.43)	(0.81 – 1.01)
3rd quintile	1.62	1.59	1.33	1.57	1.41	1.04
	(1.39 – 1.93)	(1.27 – 2.00)	(0.99 – 1.77)	(1.14 – 2.17)	(1.24 – 1.61)	(0.93 – 1.17)
4th quintile	1.71	1.69	1.71	1.21	1.43	1.09
	(1.45 – 2.01)	(1.35 – 2.12)	(1.30 – 2.26)	(0.85 – 1.70)	(1.24 – 1.64)	(0.96 – 1.24)
5th quintile	1.42	1.87	1.52	1.59	1.43	1.20
	(1.20 – 1.69)	(1.49 – 2.36)	(1.14 – 2.02)	(1.13 – 2.24)	(1.23 – 1.66)	(1.05 – 1.37)
Missing	0.60	0.61	0.68		1.10	0.58
	(0.42 – 0.85)	(0.37 – 0.99)	(0.39 – 1.18)	No missing	(0.80 – 1.50)	(0.44 – 0.76)

° Area deprivation estimates are reported in Table 9.

^1 ^Odds ratios for missing category of marital status were not estimated, because of too few individuals (n = 14).

After adjustment for all socio-demographic variables, area deprivation was significantly associated with the health outcomes when all participants were included (Table 9). The associations were more or less clear stepwise. This pattern was marked for SRH, although the confidence intervals were wide and overlapping. Compared to living in the least deprived areas, living in the most deprived areas was associated with twice as high probability for reporting poor SRH. For HSCL, CHD, smoking and no exercise we found that the deprivation quintiles 3–5 were significantly different from the first quintile. For these health outcomes, odds ratios increased by level of area deprivation, but the overlapping confidence intervals did not make us eligible to speak of any gradients in associations. For COPD, only the most deprived areas were significantly different from the least deprived areas.

**Table 9:** Odds ratios (95% CI) for associations between area deprivation and health outcomes^1^.

	**SRH**	**HSCL**	**CHD**	**COPD**	**Smoking**	**No exercise**
						
**All participants (n = 14608), year 2000.**						
1st quintile	1.00	1.00	1.00	1.00	1.00	1.00
2nd quintile	1.18	1.17	1.11	1.31	1.12	1.09
	(1.00 – 1.38)	(0.96 – 1.44)	(0.84 – 1.46)	(0.91 – 1.88)	(0.95 – 1.31)	(0.96 – 1.23)
3rd quintile	1.70	1.25	1.41	1.27	1.20	1.25
	(1.47 – 1.97)	(1.04 – 1.52)	(1.11 – 1.80)	(0.90 – 1.80)	(1.03 – 1.39)	(1.11 – 1.41)
4th quintile	1.79	1.40	1.56	1.40	1.20	1.44
	(1.56 – 2.07)	(1.17 – 1.68)	(1.23 – 1.97)	(0.99 – 1.97)	(1.03 – 1.40)	(1.27 – 1.62)
5th quintile	2.04	1.65	2.02	1.86	1.41	1.55
	(1.76 – 2.36)	(1.38 – 1.99)	(1.60 – 2.55)	(1.33 – 2.60)	(1.21 – 1.65)	(1.37 – 1.75)
						
**In-migrants removed (n = 11067), year 2000.**						
1st quintile	1.00	1.00	1.00	1.00	1.00	1.00
2nd quintile	1.23	1.22	1.24	1.25	1.12	1.10
	(1.03 – 1.47)	(0.97 – 1.54)	(0.91 – 1.68)	(0.84 – 1.86)	(0.94 – 1.34)	(0.97 – 1.26)
3rd quintile	1.69	1.24	1.51	1.31	1.22	1.27
	(1.44 – 1.99)	(1.00 – 1.54)	(1.15 – 1.98)	(0.90 – 1.89)	(1.04 – 1.44)	(1.12 – 1.44)
4th quintile	1.83	1.45	1.52	1.32	1.27	1.45
	(1.56 – 2.15)	(1.18 – 1.78)	(1.16 – 1.99)	(0.91 – 1.93)	(1.08 – 1.50)	(1.28 – 1.65)
5th quintile	2.10	1.58	2.08	1.77	1.37	1.51
	(1.78 – 2.48)	(1.27 – 1.95)	(1.59 – 2.71)	(1.22 – 2.55)	(1.16 – 1.62)	(1.32 – 1.73)
						
**In-migrants removed (n = 11067), years 1992–2000.**						
1st quintile	1.00	1.00	1.00	1.00	1.00	1.00
2nd quintile	1.27	1.21	1.41	1.28	1.14	1.14
	(1.06 – 1.52)	(0.96 – 1.53)	(1.04 – 1.92)	(0.88 – 1.86)	(0.96 – 1.34)	(1.00 – 1.30)
3rd quintile	1.84	1.20	1.90	1.27	1.32	1.32
	(1.55 – 2.19)	(0.96 – 1.50)	(1.42 – 2.53)	(0.87 – 1.84)	(1.12 – 1.56)	(1.16 – 1.51)
4th quintile	1.92	1.41	1.52	1.30	1.37	1.46
	(1.63 – 2.27)	(1.13 – 1.74)	(1.14 – 2.03)	(0.91 – 1.86)	(1.16 – 1.61)	(1.28 – 1.66)
5th quintile	2.25	1.59	2.19	1.99	1.44	1.63
	(1.90 – 2.66)	(1.28 – 1.98)	(1.66 – 2.91)	(1.40 – 2.82)	(1.22 – 1.70)	(1.42 – 1.87)

^1 ^Adjusted for all variables in Table 8.

Overall, associations between area deprivation and health outcomes were rather identical when we removed in-migrants, and when all years (1992–2000) were taken into account. For SRH, associations were slightly stronger when area deprivation was measured in 1992–2000, than in 2000. So were the associations for being in the most deprived category in 1992–2000 for CHD and COPD. None of the socio-demographic variables changed much throughout the three models (figures not shown).

In Table 10, the area deprivation quintiles were collapsed into a continuous variable, showing significant test for trend estimates in area deprivation for all six health outcomes after adjustment for individual variables. Model 1 shows that area deprivation was significantly associated with all health outcomes for all HUBRO-participants in 2000. In model 2 we excluded the in-migrants, but the test for trend estimates were practically identical to model 1, and despite a reduction in the sample size, the confidence intervals had not widened much. In model 3, taking migration within Oslo during the period 1992–2000 into account, there were no substantial changes in the area deprivation trends.

**Table 10:** Test for trend (odds ratios, 95% CI) for area deprivation° with health outcomes.

	**Model 1**	**Model 2**	**Model 3**
	**All participants**	**In-migrants removed**	**In-migrants removed**
	**2000^1^**	**2000^1^**	**1992–2000^2^**
**SRH**	1.19 (1.15 – 1.22)	1.20 (1.15 – 1.24)	1.21 (1.17 – 1.26)
**HSCL**	1.12 (1.08 – 1.17)	1.11 (1.06 – 1.17)	1.11 (1.06 – 1.17)
**CHD**	1.19 (1.13 – 1.25)	1.18 (1.11 – 1.25)	1.16 (1.09 – 1.24)
**COPD**	1.14 (1.05 – 1.24)	1.13 (1.03 – 1.23)	1.16 (1.06 – 1.26)
**Smoking**	1.08 (1.04 – 1.11)	1.08 (1.03 – 1.12)	1.09 (1.05 – 1.13)
**No exercise**	1.12 (1.09 – 1.15)	1.11 (1.08 – 1.15)	1.13 (1.09 – 1.16)

° Adjusted for all individual variables in Table 8.

^1 ^Deprivation measured according to residence in year 2000.

^2 ^Deprivation measured as an average during the period 1992–2000.

## Discussion

This study suggests that cross-sectional studies are weakly biased by misclassification of area exposure due to recent migration (Tables 9 and 10). The strong associations between area deprivation and health outcomes that we found after adjustment for individual variables (Table 8) were not changed when we removed those study participants that had moved into Oslo during the last nine years prior to the study, or when we took all nine years of area deprivation into account for those who had lived in Oslo in all years. Contrary to our expectations, this study has shown that the associations between area deprivation and health are basically identical in analyses that do and do not take recent migration into account.

Why did we not find any substantial differences between models where all participants were included, and models where in-migrants were removed? The in-migrants constituted a very young age group, which is in line with previous studies demonstrating that young adulthood is the peak age for migration, when people tend to move into urban areas for education and employment opportunities [1,35]. Age-standardised prevalences of health outcomes were about the same for in-migrants and those who had lived in Oslo. Because the in-migrants were evenly distributed across the area deprivation quintiles, they did not affect those estimates that would have been obtained if only those who lived in Oslo in 1992–2000 were included (Table 9, model 2).

When we compared those who had lived all years in Oslo measured according to their place of residence in year 2000 (Table 9, model 2) and residence for all nine years (Table 9, model 3) we also found weak differences. We assumed that model 3 would give a more accurate measure of area deprivation exposure due to, *firstly*, a longer time-span that would better take into account movings caused by recent life events such as a divorce, which is a factor shown to trigger moving [47]. *Secondly*, because the changes in area deprivation during the period were taken into account, which has been proven to be important [6], but not so in our study due to a remarkable stability of the areas in the deprivation hierarchy (Table 2). Although there were changes during the period in the five items that constituted our composite deprivation measure, they did not change the areas' positioning in the deprivation hierarchy. *Thirdly*, because the possibility of misclassification of exposure was substantially reduced, as all movings within Oslo during nine years were registered. But although 45% of those who lived all years in Oslo were assigned different area deprivation categories in 1992 and 2000, most movings had been only one category up or one category down (Table 4). Hence, the average area deprivation scores over nine years, assigned to all individuals in model 3, did not differ much from the cross-sectionally assigned values in model 2 based on current residence (Table 9), illustrated by a correlation of .862 (p < .01).

We did not find significant area variance in all health outcomes after adjusting for the independent socio-demographic variables (Table 7). Yet, we found that area deprivation was significantly associated with all health outcomes. This is not a contra intuitive fact [48]. Methodological explanations for this are discussed in detail elsewhere [49]. In short, such findings may result from two main sources. *Firstly*, regarding the number of areas, the power to detect the variance components is affected by the number of groups and the number of persons per group in a different way than the power to detect the effects of a specific area level variable on an individual health outcome. Thus, a given study may have insufficient power to detect between area variance, and yet have sufficient power to detect the effects of a specific area variable [49], because the area variance depends more on the number of areas than on the number of people [37,50]. Data availability confined us to use 25 areas that were big and heterogeneous and clearly not in line with the boundaries that shape the relevant environment for a specific individual health outcome [50]. If data had allowed us to break down the areas into smaller units, we would most likely have seen a more pronounced variability between the areas because they would have become more homogeneous [51], as in e.g. Reijneveld et al. [52] where administrative areas split into smaller units gave increased area variances in health outcomes. *Secondly*, separating compositional from contextual effects is difficult [49], and if there is anything such as area contexts causally related to health, the pathways involved are likely to be complex and involve reciprocal causation and feedback loops [49,53,54]. There may be cross-level interaction effects present, in which the area-level impacts work through the individual-level variables and do not appear in the area-level variance [55]. Therefore, Diez Roux [49] suggests that the only way to test a specific hypothesis about an area effect is to explicitly test it rather than rely on estimates of area-level variance to determine whether or not the hypothesis is worth testing.

### Study strengths and limitations

The Oslo Health Study had a response rate of only 46%. There were also many missing values on our independent variables (Table 1). The representativeness of the participants in the analyses is debatable, but a highly representative sample is no longer considered essential for generalisability in etiological studies that report risk estimates rather than prevalence estimates [36]. However, our primary aim was to compare the results of a cross-sectional study (Table 9, model 1) with the results when migration was accounted for in a quasi longitudinal design (Table 9, models 2 and 3), thus making the representativeness of the participants a subordinate issue.

The choice and size of area borders is a much discussed issue [52,56,57]. Administrative areas may not be ecologically meaningful or natural [25,58-60]. The choice of areas in this study was driven by available deprivation data. The large sizes of our administrative areas (mean number of inhabitants was 20148) may have been too large to capture true associations between health and areas [61]. According to Reijneveld et al[52] it is a common view that smaller areas should result in a more valid or more stable measurement of area deprivation. The use of smaller areas may lead to an increase in measurement error, but small areas will at the same time be more homogeneous in terms of their socioeconomic and other important characteristics [62]. However, some studies have found that administrative areas and smaller local areas provide similar results. Reijneveld et al[52] used three types of area classifications for Amsterdam: neighbourhoods (mean: 7 850 inhabitants), postcode sectors (mean: 9 504) and boroughs (mean 32828), and found that health differences by area deprivation differed only slightly for the three geographical classifications, both with and without adjustment for individual level socioeconomic variables. Similarly, in a study from Montreal, Ross et al[56] found that when 28 administrative areas were split into 118 'natural neighbourhoods', the models using administrative areas had remarkably similar results to the 'natural neighbourhoods', suggesting that administrative areas were good proxies for natural neighbourhoods.

Still, within our framework, it is possible that the heterogeneity of our areas (i.e. they may include both deprived and less deprived smaller neighbourhoods) leads to misclassification of exposure. It is of course fully possible to move from the most deprived local neighbourhood in a non-deprived area, to the least deprived local neighbourhood in a deprived area. Answering which of those areas that is in fact most deprived is complicated.

Our data did not allow us to specifically test such issues, and it remains a potential pitfall. Some recent studies have found that the association between area deprivation and health operate on a much smaller scale than that of administrative areas, by using spatial techniques with circular areas centered on the exact place of residence of individuals [61,63,64]. Such techniques also deal with the problem of people residing on the margins of an administrative area, for whom an assigned 'area status' may be rather artificial. Yet, despite the shortcomings of large administrative areas, significant associations were found.

Our effort of assigning area deprivation indexes for all years proved not be worthwhile as the areas did not change their deprivation status during the years. We cannot rule out the possibility that this stability was a result of our chosen deprivation indicators. If other indicators had been chosen, then there might have been strong enough changes in those indicators during the period 1992–2000 to change the areas' positioning in the deprivation hierarchy. Another weakness of the present study is that our health outcomes are self-reported with relatively simple instruments.

There are several strengths to this study. We did investigate to what extent the associations between area deprivation and health were confounded by smoking, which is a much debated issue [18,65]. Adding smoking as an independent variable to our models, showed a strong and significant association between smoking and the health outcomes (figures not shown), but adding smoking had no impact on the associations between area deprivation and health outcomes. This has also been found in previous studies on e.g. CHD [66,67]. Furthermore, our results were adequately controlled for confounding by socioeconomic position [68], and it is unlikely that incomplete adjustment for individual status is the reason why we found associations between area deprivation and health [31].

Although we could not find much difference between models with all participants and models where migrants were excluded, we did control for this, i.e. migration within Oslo and migration into Oslo, which is rarely done in cross-sectional studies. One aspect of migration that we could not adequately control for, was that of selective migration. Evidence from international studies are few, and with somewhat contradictory results. In a recent Dutch study, van Lenthe et al[43] found little support of selective migration, whereas Norman et al[1] found that over a 20-year period, migrants who moved from more to less deprived areas were healthier than migrants who moved from less to more deprived areas, implying that selective migration exaggerated the association between area deprivation and health.

Our cross-sectional design did not allow us to investigate this, but we did investigate differences in characteristics between those who moved upwards and downwards in Oslo (unfortunately, the in-migrants could not be included in this analysis, as deprivation data for areas outside of Oslo was not available). Those who moved up were older, better educated, had higher income and better self rated health compared to those who moved down (Table 5). But after adjusting for all individual variables, those who moved down could not be claimed to have worse health than those who moved up (Table 6). Interestingly, in the same regression analysis, we found that migration within Oslo, regardless of direction, was significantly associated with worse health compared to residing in the same area deprivation category. Within the limitations of a cross-sectional design, this does not lend support to the hypothesis that selective migration might account for the association between area deprivation and health.

## Conclusion

From this study we outline one empirical and one methodological conclusion. Empirically, we have documented strong and significant associations between area deprivation and health outcomes in Oslo after adjustment for individual socio-demographic variables. Methodologically, we have demonstrated that these associations were weakly biased by recent migration, a commonly considered, but unresolved problem in cross-sectional analyses. Associations between area deprivation and health outcomes varied in a minor way between models that took recent migration into account and models that did not.

## Competing interests

The author(s) declare that they have no competing interests.

## Authors' contributions

FNP planned the study design, performed the analyses and wrote the manuscript. BC and ØN participated in planning the study design, contributed with academic discussions and drafted and revised the manuscript. All authors read and approved the final manuscript.
